# Temporal complexity in photoplethysmography and its influence on blood pressure

**DOI:** 10.3389/fphys.2023.1187561

**Published:** 2023-08-31

**Authors:** Xiaoman Xing, Rui Huang, Liling Hao, Chenyu Jiang, Wen-Fei Dong

**Affiliations:** ^1^ School of Biomedical Engineering, Division of Life Sciences and Medicine, University of Science and Technology of China, Suzhou, China; ^2^ Suzhou Institute of Biomedical Engineering and Technology, Chinese Academy of Sciences, Suzhou, China; ^3^ Academy for Engineering and Technology, Fudan University, Shanghai, China; ^4^ College of Medicine and Biological Information Engineering, Northeastern University, Shenyang, China; ^5^ Jinan Guoke Medical Technology Development Co. Ltd., Jinan, China; ^6^ Suzhou GK Medtech Science and Technology Development (Group) Co. Ltd., Suzhou, China

**Keywords:** photoplethysmography, blood pressure, single-site, Windkessel model, temporal patterns

## Abstract

**Objective:** The temporal complexity of photoplethysmography (PPG) provides valuable information about blood pressure (BP). In this study, we aim to interpret the stochastic PPG patterns with a model-based simulation, which may help optimize the BP estimation algorithms.

**Methods:** The classic four-element Windkessel model is adapted in this study to incorporate BP-dependent compliance profiles. Simulations are performed to generate PPG responses to pulse and continuous stimuli at various timescales, aiming to mimic sudden or gradual hemodynamic changes observed in real-life scenarios. To quantify the temporal complexity of PPG, we utilize the Higuchi fractal dimension (HFD) and autocorrelation function (ACF). These measures provide insights into the intricate temporal patterns exhibited by PPG. To validate the simulation results, continuous recordings of BP, PPG, and stroke volume from 40 healthy subjects were used.

**Results:** Pulse simulations showed that central vascular compliance variation during a cardiac cycle, peripheral resistance, and cardiac output (CO) collectively contributed to the time delay, amplitude overshoot, and phase shift of PPG responses. Continuous simulations showed that the PPG complexity could be generated by random stimuli, which were subsequently influenced by the autocorrelation patterns of the stimuli. Importantly, the relationship between complexity and hemodynamics as predicted by our model aligned well with the experimental analysis. HFD and ACF had significant contributions to BP, displaying stability even in the presence of high CO fluctuations. In contrast, morphological features exhibited reduced contribution in unstable hemodynamic conditions.

**Conclusion:** Temporal complexity patterns are essential to single-site PPG-based BP estimation. Understanding the physiological implications of these patterns can aid in the development of algorithms with clear interpretability and optimal structures.

## 1 Introduction

Blood pressure (BP) is one of the most important vital signs and is closely related to the prognosis of cardiovascular disease, which ranks first in all-cause mortality (Chobanian et al., 2003). Although the office blood pressure measurement (OBPM) is still the recommended diagnostic tool, ambulatory blood pressure monitoring (ABPM) can offer more details about BP fluctuation and help improve the diagnosis (Force et al., 2021). A lightweight and easy-to-use ambulatory BP monitor could help promote the long-term management of hypertension (Agarwal et al., 2011).

The use of photoplethysmography (PPG) for estimating BP has gained popularity in recent years due to its affordability and convenience (Martínez et al., 2018; Elgendi et al., 2019; Josep Solà and Josep Solà, 2019; Cosoli et al., 2020). However, several drawbacks prevented its widespread usage. The first problem is that current theoretical models may lead to unstable BP prediction in practice. The most well-known pulse transition time (PTT) methods assumed correlations between PTT and arterial compliance (*C*) (Mukkamala et al., 2015; Ding et al., 2016; Mukkamala and Hahn, 2018). But cardiac output (CO) and peripheral resistance (*R*) to blood flow also had considerable contributions to BP changes. Calibrations must be done frequently, and sudden failures may occur (Butlin et al., 2018; Finnegan et al., 2021; Avolio et al., 2022). Another theoretical proposal used the four-element Windkessel (WK4) model to estimate major lump hemodynamic properties (Wang et al., 2017; Xing et al., 2021), which could be used to stabilize the measurement. However, this model only used PPG morphological features, making it susceptible to environmental disturbances such as contact pressure, sensor placement, and temperature fluctuations (Hsiu et al., 2011; Hsiu et al., 2012; Grabovskis et al., 2013). Therefore, obtaining reliable BP estimations from PPG morphology alone, even after calibration, remains challenging (Xing et al., 2019; Hosanee et al., 2020). Another problem with single-site PPG-derived BP is associated with instability in ambulatory measurement. Most of the studies required the subjects to stay motionless in a supine or sitting position. The performance of BP estimation may deteriorate quickly in motion because PTT and PPG morphology are sensitive to noise and posture changes (Allen and Murray, 1999; Pour Ebrahim et al., 2019).

Although the “black-box” encoding in machine learning algorithms lacks clear interpretability, they have achieved remarkable performance in practice. Some were deployed in continuous BP measurement and showed improvement in both accuracy and stability (Radha et al., 2019; El-Hajj and Kyriacou, 2021; Yen et al., 2021). Most of them used time-dependent information, such as the long- and short-term memory (LSTM) network (Monte-Moreno, 2011; Radha et al., 2019; Harfiya et al., 2021; Li et al., 2021; Pu et al., 2021; Wang et al., 2021; Ali and Atef, 2022; Meng et al., 2022), system identification (Allen and Murray, 1999), auto-regression (Acciaroli, 2018), multi-stage feature extraction (Ali and Atef, 2022; Jiang et al., 2022), dynamic compliance (Gupta et al., 2022), or simple heart rate variability(HRV) (Mejía-Mejía et al., 2022). These algorithms performed better than those without dynamic features (Radha et al., 2019; Harfiya et al., 2021). In this study, we aim to give a plausible explanation of the system’s temporal complexity. With this knowledge, optimizing the structure of the machine learning algorithms would be easier.

Physiological model-based PPG simulations may help decode this “black box”. However, current PPG synthesis methods have limitations, with some being overly complicated (Charlton et al., 2019; Mazumder et al., 2022) and others overly simplistic (Tang et al., 2020a; Tang et al., 2020b). The complex physiological models often require human anatomical data and intricate coupling between vascular segments. While they serve as excellent approximations of real PPG signals and are valuable for disease diagnosis, studying rapid hemodynamic changes becomes challenging due to their high computational cost. On the other hand, simple PPG synthesis models combine forward and reflected waves, aiding in PPG event detection. However, these models lack essential hemodynamic details, such as compliance dependent on BP (Tang et al., 2020a). In this study, our aim is to develop a user-friendly simulation tool by modifying the classic four-element Windkessel model. By updating the simulation per heartbeat, we can generate stimuli with varying timescales and observe subsequent PPG responses. This approach enables the simulation of fast-changing CO, *R*, and compliance, allowing us to investigate unstable hemodynamic conditions. Through this simulation tool, we can gain insights into the stochastic behavior of PPG and evaluate their potential contribution to BP estimation.

This study introduces several key novelties and findings, including.

(1) A novel *in silico* simulation method is proposed to generate dynamic PPG signals with time- and BP-dependent compliance profiles.
(2) The variation of central vascular compliance (*C*_1_) throughout a cardiac cycle, along with CO and *R*, collectively determine the time delay, amplitude overshoot, and phase lag of the PPG response to a pulse stimulus.
(3) Continuous simulations showed that complicated temporal PPG patterns could be generated by random stimuli, which means that the “passive” buildup of phase lags and amplitude fluctuations are related to hemodynamic fluctuations.
(4) The complexity of stimuli directly influences the complexity of the resulting signal.
(5) The addition of temporal complexity features increased the stability and accuracy of BP estimation, especially at high CO fluctuations.

The rest of this paper is organized as follows. Section 2 provides a detailed description of the modified four-element Windkessel model, pulse and continuous simulation procedures, experimental dataset, validation procedure, and complexity measures. Section 3 presented the simulation results and the complexity feature distribution, which were validated using multi-modal experimental data. The contribution of complexity and morphological features to BP estimation under stable and unstable cardiac conditions were calculated and compared. Section 4 discussed the physiological implication of these findings and the potential advantages of using complexity features in BP estimation. Section 5 concludes the paper.

## 2 Materials and methods

**FIGURE 1:**
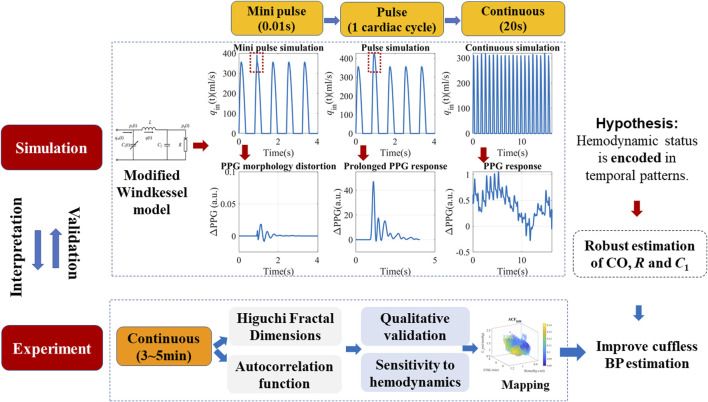
Schematics of the proposed simulation method, hypothesis, and experimental verifications. The modified Windkessel model is introduced in detail in Section 2.1, with a simplified assumption of the left ventricular ejection (*q*_in_). Pulse and continuous simulations with different timescales were carried out to locate the origin of complexity patterns.

This section mainly describes the *in silico* simulation and experimental verification procedures, as illustrated in Figure 1. For simulation, a modified WK4 model with BP-dependent compliance was introduced. PPG responses to both pulse and continuous stimuli at various timescales were simulated and quantified. Temporal complexity and correlation measures such as HFD and ACF are proposed to describe the PPG responses. Their contribution to BP was calculated and compared to morphological features. Multi-modal continuous experimental recordings were used to verify the simulation results.

### 2.1 The modified WK4 model with time- and BP-dependent compliance

**FIGURE 2:**
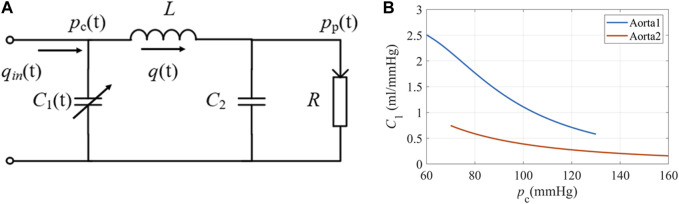
**(A)** The equivalent circuit of the modified WK4 model. *q*(t) represents blood flow. *C*_1_ is time-dependent and varies with central BP. **(B)** The elastic property of the aorta under different pressures (Langewouters et al., 1984). Aorta1: Am = 3.5 cm^2^, P0 = 50.4 mmHg, P1 = 42.3 mmHg; Aorta2: Am = 6.18 cm^2^, P0 = −2.3 mmHg, P1 = 21.6 mmHg.

To get a rough idea of the PPG response to stimuli, we did a series of simulations using a modified WK4 model with time- and BP-dependent compliance, as shown in Figure 2A. In this model, the heart is represented as a current source *q*_in_. The arterial tree system is modeled by four major parameters. Unlike traditional WK4 models (Wang et al., 2017), *C*_1_ is designed to vary within a cardiac cycle, which depends on central BP (*p*_c_), as shown in Figure 2B. *R* reflects the peripheral resistance, which mainly comes from small arteries, arterioles, and capillaries. BP changes induce corresponding arteriolar resistance changes to keep capillary pressure constant and maintain tissue fluid equilibrium (Nicolaas Westerhof and Noble, 2010). Although *R* also varies during a cardiac cycle, the overall fluctuation is smaller during a heartbeat. Thus, *R* is treated as a constant or slowly changing parameter. To reduce the complexity of the model, compliance of the distal arteries (*C*_2_), and inertance (*L*) are set to be time-invariant, which are added to increase the PPG waveform fitting accuracy (Westerhof et al., 2009). The blood pressure at the peripheral site (*p*_p_) could be obtained if the cardiovascular and hemodynamic parameters are known. Since the amplitude of PPG depends on the tissue substrate, microvasculature, and the coupling coefficient of the sensor and skin, personalized transfer functions were considered to convert BP to PPG (Millasseau et al., 2000).

In the time-dependent WK4 model, *C*_1_(t), *p*_c_(t), and *p*_p_(t) are strongly interdependent, as shown in equations (1a)–(1c).

$$\left\{\begin{array}{c} \frac{dq\left(t\right)}{dt}=\frac{1}{L}\left(p_{c}\left(t\right)-p_{p}\left(t\right)\right)\ldots \ldots \ldots \left(1a\right)\\ \frac{dp_{c}\left(t\right)}{dt}=\frac{1}{C_{1}\left(t\right)}\left(q_{in}\left(t\right)-q\left(t\right)\right)\ldots \ldots \ldots \left(1b\right)\\ \frac{dp_{p}\left(t\right)}{dt}=\frac{1}{C_{2}}\left(q\left(t\right)-\frac{p_{p}\left(t\right)}{R}\right)\ldots \ldots \ldots \left(1c\right) \end{array}\right.$$ (1)

In this study, *q*_in_(t) is assumed to have the flow profile described by Equation (2), where *q*_0_ is the maximum *q*_in_(t). *T*_s_ is the left ventricle ejection duration and *T* is the cardiac cycle; *α* determines the peak time of *q*_in_(t), which is set as 1/3. Note that it is a simplified approximation used to facilitate the simulation. For a more realistic simulation, *q*_in_(t) could be replaced by a numeric array obtained by *in-vivo* experiments. *q*_0_ is closely related to stroke volume (SV) and CO, as the integral of *q*_in_(t) within a cardiac cycle gives SV. Multiplied by heart rate and we can obtain CO.

$$q_{in}\left(t\right)=\left\{\begin{array}{cc} q_{0}\sin\left(\frac{\pi t}{2\alpha T_{s}}\right) & \left(0\leq t\leq \alpha T_{s}\right)\\ q_{0}\cos\left(\frac{\pi t}{4\alpha T_{s}}\left(t-\alpha T_{s}\right)\right) & \left(\alpha T<t\leq T_{s}\right)\\ 0 & \left(T_{s}<t\leq T\right) \end{array}\right.$$ (2)

*C*_1_(t) oscillates in a wide range for subjects with elastic blood vessels (Hallock and Benson, 1937). Langewouters *et al.* proposed a model to describe the cross-sectional compliance of the aorta, which used three independent parameters (Langewouters et al., 1984). In this study, we used volume compliance. *C*_1_(t) is modified by adding a unit length *l*, as shown in Equation 3.

$$C_{1}\left(t\right)=\frac{A_{m}l}{\pi P_{1}\left[1+{\left(\left(p_{c}\left(t\right)-P_{0}\right)/P_{1}\right)}^{2}\right]}$$ (3)

A_m_ is the maximum cross-sectional area of the aorta, and P_0_ is the transmural pressure when compliance reached its maximum. P_1_ represents the steepness of the compliance rise. We chose two representative sets of values from the published dataset and illustrated the compliance-pressure relationship in Figure 2B.

Our ultimate goal is to estimate *p*_p_(t) and generate corresponding PPG signals. By analyzing Eqs. 1–3, we found that they could be combined to yield a differential equation with only one unknown variable. The procedure is as follows:

(1) Combine Eqs 1a–1c and eliminate *p*_c_(t) and *q*(t). The resulting equation has only two unknown variables *C*_1_(t) and *p*_p_(t), as shown in Equation (4). All the other parameters were assumed to be known.

$$\frac{d^{3}p_{p}\left(t\right)}{dt^{3}}+\frac{1}{RC_{2}}\frac{d^{2}p_{p}\left(t\right)}{dt^{2}}+\left(\frac{1}{LC_{1}\left(t\right)}+\frac{1}{LC_{2}}\right)\frac{dp_{p}\left(t\right)}{dt}+\frac{1}{LRC_{1}\left(t\right)C_{2}}p_{p}\left(t\right)=\frac{1}{LC_{1}\left(t\right)C_{2}}q_{in}\left(t\right)$$ (4)

(2) Combining Equations (1a), (1c), we could obtain Equation (5) by eliminating *q*(t). Then *C*_1_(t) in Equation (3) becomes an expression that depends solely on variable *p*_p_(t), as shown in Equation (6).

$$p_{c}\left(t\right)=\left(\frac{d^{2}p_{p}\left(t\right)}{{dt}^{2}}+\frac{1}{C_{2}R}\times \frac{dp_{p}\left(t\right)}{dt}\right)C_{2}L+p_{p}\left(t\right)$$ (5)

$$C_{1}\left(t\right)=\frac{A_{m}l}{\pi P_{1}\left[1+{\left(\frac{\left[\left(\frac{d^{2}p_{p}\left(t\right)}{{dt}^{2}}+\frac{1}{C_{2}R}\times \frac{dp_{p}\left(t\right)}{dt}\right)C_{2}L+p_{p}\left(t\right)-P_{0}\right]}{P_{1}}\right)}^{2}\right]}$$ (6)

(3) By replacing *C*_1_(t) in Equation (4) with the expression in Equation (6), the resulting differential equation has only one unknown variable *p*_p_(t), which could be solved with the Runge-Kutta (4,5) formula (ODE45) in Matlab 2021b.
(4) When *p*_p_(t) is obtained, PPG could be subsequently calculated by using the *P-V* relationship (Millasseau et al., 2000). For this study, we conducted simulations with a time resolution of 0.01 s. Although a higher resolution could potentially handle more complex *q*_in_(t) profiles, a step size of 0.01 s is sufficient given that we primarily employed an analytical description of *q*_in_(t).

Although the modified WK4 model provided realistic PPG waveforms, caution should be taken to interpret the simulation results. Firstly, the original WK4 model was proposed to explain the formation of peripheral BP waveforms at different frequencies (Nicolaas Westerhof and Noble, 2010). The hemodynamic parameters must be constant to yield a reasonable impedance explanation. In this study, we let the compliance be time-dependent, which is physiologically sound, but the simulation results should not be used to modify the characteristic impedance. Secondly, the simulated PPG waveforms may deviate from *in-vivo* measurements due to the oversimplified *q*_in_(t) and cardiovascular system. This modified time-dependent WK4 model is used to qualitatively explore the stochastic patterns of PPG, which must be verified using experimental data. Thirdly, WK4 is an open-loop model, which assumes that *q*_in_(t) is known and does not depend on cardiovascular feedback. For a more realistic model, the influence on *q*_in_(t) should be considered to form a more complex closed-loop model. As a result, the change in blood flow can have much longer impact than an open-loop model.

Considering the complexity of the topic, we have chosen to commence our study with simpler models before gradually advancing to more intricate and realistic ones. By initially employing an open-loop model, we were able to establish a preliminary dynamic relationship between PPG and BP. This approach not only facilitates easier comprehension but also enhances safety during the research process.

To sum up, the *in silico* simulation provided guidance, while experimental data must be used to refine the details.

### 2.2 Experimental data

To generate realistic simulation data, we used some of the experimentally measured data as the model input. For example, we used 45 different *C*_1_ profiles from Langewouters *et al* (Langewouters et al., 1984). Multi-modal continuous vital recordings are from a publicly available database by Charles Carlson et al. (Carlson et al., 2020), which consists of short PPG-BP measurements from 40 healthy subjects. The original study involving human participants was reviewed and approved by the Kansas State University Institutional Review Board (protocol number 9386, approved 5 July 2019). Informed consent was obtained from all subjects involved in the study. The more popular Medical Information Mart for Intensive Care (MIMIC) database is not used due to its lack of PPG amplitude information (Johnson et al., 2016).

The subjects took a supine position and each measurement lasted for around 5 min. Finger PPG was acquired using a GE patient monitor (Datex CardioCap 5). Continuous brachial BP waveforms and stroke volume (SV) were derived from Finometer PRO (Finapres Medical Systems). The raw data were resampled to 100 Hz and lowpass filtered with a cut-off frequency of 10 Hz. For temporal pattern calculation, data with sufficient length is required. We used a window of 20 s and moved one cardiac cycle each time to increase the sample size. A total of 17,476 measurements were obtained from 40 subjects. The subjects’ demographics are listed in Table 1.

**TABLE 1:** Subjects’ demographics.

Number of subjects	Age (years)	Height (cm)	Weight (kg)	BMI (kg/m^2^)	Sex (M/F)	SBP (mmHg)	DBP (mmHg)
40	34 ± 15	171 ± 11	76 ± 18	26 ± 5.7	17/23	120 ± 14	69 ± 13

* SBP: systolic BP; DBP: diastolic BP.

The hemodynamic status of each measurement is estimated to help understand the underlying physiological mechanism. N Stergiopulos *et al.* found that *C*_1_ and *R* could be accurately estimated by the Windkessel model (Stergiopulos et al., 1994; Nicolaas Westerhof and Noble, 2010). In this study, we used a similar approach to estimate *C*_1_ and *R* (Xing et al., 2021), except that *C*_1_ had to be chosen from the 45 published profiles, as in Equation (3) (Langewouters et al., 1984). We performed individual test on each subject and each *C*_1_ profile using the time-dependent Windkessel models, taking into account the variability of BP within the cardiac cycle. For each pair, we adjusted *R*, *L*, and *C*_2_ to minimize the discrepancy between the simulated and measured BP waveforms. On a per-subject basis, the *C*_1_ profile that exhibited the best match (as indicated by the lowest root mean square error, RMSE) to the measured BP waveform was selected. Alternative approaches were used to ensure the validity of hemodynamic estimation. For example, *R* is also estimated by calculating the ratio of the mean arterial pressure (MAP) and CO (Nicolaas Westerhof and Noble, 2010). *C*_1_ is also estimated by calculating the ratio of the peak-to-peak PPG amplitude and the pulse pressure (PP) of BP (Allen and Murray, 1999). We found that the Windkessel model derived *C*_1_ and *R* linearly correlated with the alternative methods in this dataset. If different models yielded considerably different estimations, we discard the corresponding samples.

### 2.3 Pulse stimuli and the corresponding PPG responses

**FIGURE 3:**
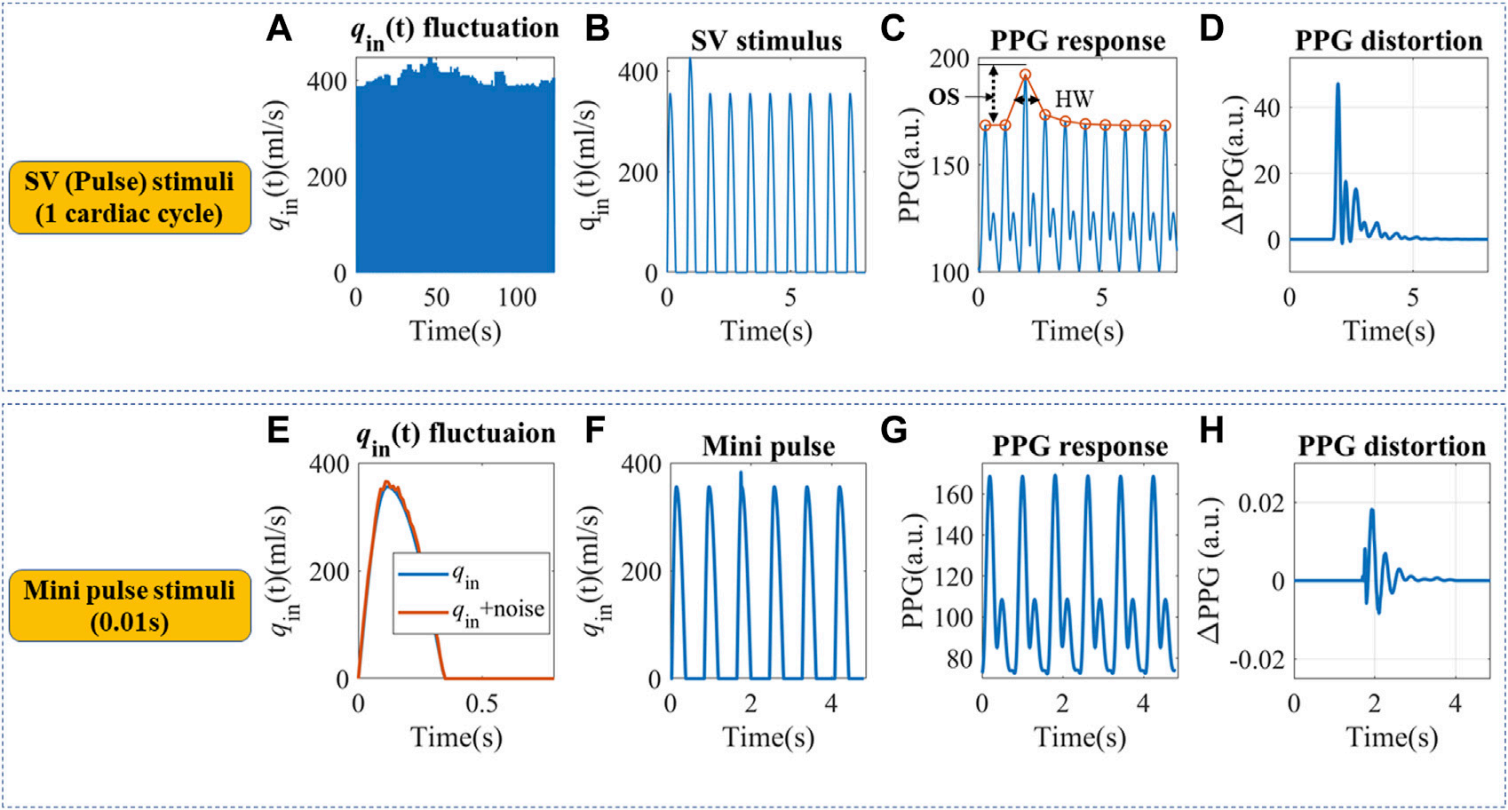
**(A)** Continuous *q*_in_ fluctuation with SV variation. **(B)** Definition of an SV pulse stimulus that lasted a cardiac cycle. **(C)** The simulated PPG response and definition of half width (HW) and overshoot (OS). Simulation parameters were set as follows: A_m_ = 3.5 cm^2^, P_0_ = 50.4 mmHg, P_1_ = 42.3 mmHg, *T* = 0.8 s, α = 1/3, *T*_s_ = 0.35 s, CO = 5.95 L/min, *C*_2_ = 0.1 mL/mmHg, *R* = 1.4 mmHg s/ml, *L* = 0.03 mmHg s^2^/ml. **(D)** The SV pulse-induced PPG changes. **(E)** *q*_in_ fluctuation within a cardiac cycle. **(F)** Definition of a “mini” pulse stimulus that lasted for 0.01 s. **(G)** The simulated PPG response to a “mini” pulse. **(H)** The “mini” pulse induced PPG changes.

Real hemodynamic stimuli are complicated, as shown in Figures 3A,E. However, they could be decomposed into continuous pulse simulations with different timescales, which may help to understand the physiological mechanism. We designed pulse *in silico* simulations with long and short durations to investigate the corresponding PPG responses, as shown in Figures 3B,F. Firstly, a simulation with a 20% *q*_0_ increase that lasted a single cardiac cycle was built, hereby referred to as SV stimuli. It is the easiest to model, understand and quantify. Then very short stimuli with a 10% increase of *q*_in_(t) that lasted for 0.01s was simulated, hereby referred to as “mini” stimuli.

For the *in silico* SV stimuli simulation, cardiovascular systems with different *C*_1_ and *R* were used. Two indices were proposed to describe the recovery time from a single stimulus, as shown in Figure 3C. Half width (HW) is defined as the width at 50% of the peak response, which measures the recovery time for a single stimulus. Longer HW may lead to overlaps of responses and complex signal fractal structures. The height of the overshoot (OS) is associated with the maximum magnitude of BP or PPG fluctuations. The PPG distortions are defined as the difference between PPG signals with and without stimuli, as illustrated in Figures 3D,H. “Mini” stimuli caused similar but much smaller amplitude responses (∼10^−3^ of the response from SV stimuli), which were more pronounced in the derivatives of the PPG signal.

To gain a thorough understanding of the time-dependent PPG responses to stimuli, we used all the 45 *C*_1_ profiles from the published dataset (Langewouters et al., 1984) and varied the peripheral resistance from 0.7 to 1.3 mmHg s/ml, with a step size of 0.2 mmHg s/ml. Three CO levels were tested at 4.25L/min, 5.1 L/min, and 5.95 L/min. Simulations with SBP higher than 220 mmHg or lower than 80 mmHg were discarded, since these out-of-range SBPs did not match our experimental data and may potentially deviate from the simplified model.

The result from SV stimuli simulations could be extrapolated to more complicated situations. For example, *q*_in_ contour irregularity could also be decomposed into an infinite number of “mini” stimuli (Nicolaas Westerhof and Noble, 2010), similar to Figures 3E,F. These “mini” stimuli mainly influence the *C*_1_ trajectory during a cardiac cycle and lead to small phase shifts, as in Figures 3G,H. We assumed PPG responses to “mini” stimuli were similar to SV stimuli, but at a smaller scale.

### 2.4 Continuous stimulation and experimental validation

#### 2.4.1 Continuous stimulation: Random stimuli

In a real-life application, the cardiovascular system constantly adjusts CO, heart rate (HR), *C*_1_, *R*, and *q*_in_ contour depending on the metabolic need and hemodynamic feedback from the entire body. In addition, autocorrelation patterns can also be observed in the fluctuations of SV and *R*, as depicted in Supplementary Figures S1, S2, which may lead to longer cardiovascular responses. The accumulated PPG responses may form long- and short-term temporal patterns. For simplicity, we chose CO and *R* perturbation to study longer-term complexities, and assumed *q*_in_-caused short-term complexities have similar behavior. To isolate the origin of complexity, random stimuli were used. Peripheral BP signals (*p*_p_(t)) were generated by the modified WK4 model, and PPG is translated from BP by personalized pressure-volume translations. Each simulation contained 20 cardiac cycles.

**FIGURE 4:**
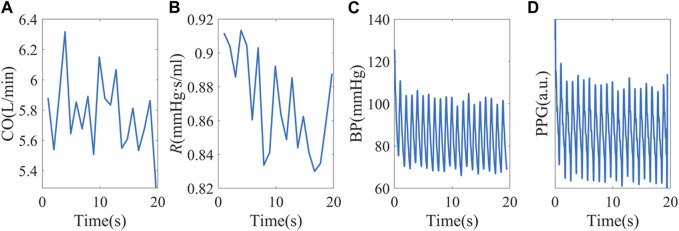
**(A, B)** Random CO and *R* stimuli using baseline hemodynamic status from subject X1047. **(C)** Simulated peripheral BP. **(D)** PPG signals were obtained from personalized *P-V* translations.

To build a more realistic simulation, the hemodynamic status of the 40 healthy subjects was estimated and used as simulation inputs. The mean CO and *R* of each subject were used as baselines, and random perturbations were added. We used a white noise randomly chosen from −5% to 5% of the baseline, with a mean of 0 and standard deviation of 2.83%. To increase sample sizes, CO and *R* baselines were also shifted by ±10%. An example simulation is shown in Figure 4. This test is to investigate the possibility of forming complexity patterns just from “passive” hemodynamic responses.

In real-life scenarios, stimuli like SV and *R* may exhibit autocorrelation owing to cardiovascular auto-regulation, as demonstrated in Supplementary Figures S1, S2. The experimental BP and PPG complexity measures exhibit combined impact of the stimuli and vascular response.

#### 2.4.2 Experimental validation: Distribution map and contribution evaluation

For experimental validation, complexity measures such as HFD and ACF were calculated for each 20s sampling window, and their correlation with hemodynamic status is used to investigate the agreement between model prediction and experimental data. For practical usage, three-dimensional maps of complexity measures and their gradients were generated for experimental data.

Furthermore, to assess the influence of temporal patterns to BP under stable and unstable cardiac conditions, we calculated the Pearson correlation coefficient (PCC) of each temporal feature and BP. Additionally, we employed Bayesian neural network to evaluate their collective nonlinear contributions, as explained in Section 2.7. Comparisons were made with commonly-used single-site morphological features, as defined in Table 2 and documented in Supplementary Figure S3 (Elgendi, 2012). Biometric input was not permitted to prevent information leakage. In order to maintain consistency with temporal features, we performed a median averaging of the morphological features, resulting in one reading per 20-s epoch. This approach enables us to simultaneously evaluate the contributions from both morphological and temporal features. By considering these aspects together, we gain a comprehensive understanding of the characteristics under analysis.

**TABLE 2:** Definition of selected morphological features.

Features	Definition
AC	The pulsating amplitude of PPG
DC	Mean of PPG baseline
Area	The area under the normalized PPG waveform
Notch index (NI)	Notch index. The waveform value at the dicrotic notch over the systolic peak
SPMEAN	Mean upstroke slope during the systolic period
SPVAR	Variation of upstroke slope (standard deviation) during the systolic period
DPMEAN	Mean downstroke slope during the diastolic period
DPVAR	Variation of downstroke slope (standard deviation) during the diastolic period

### 2.5 Complexity measures

**FIGURE 5:**
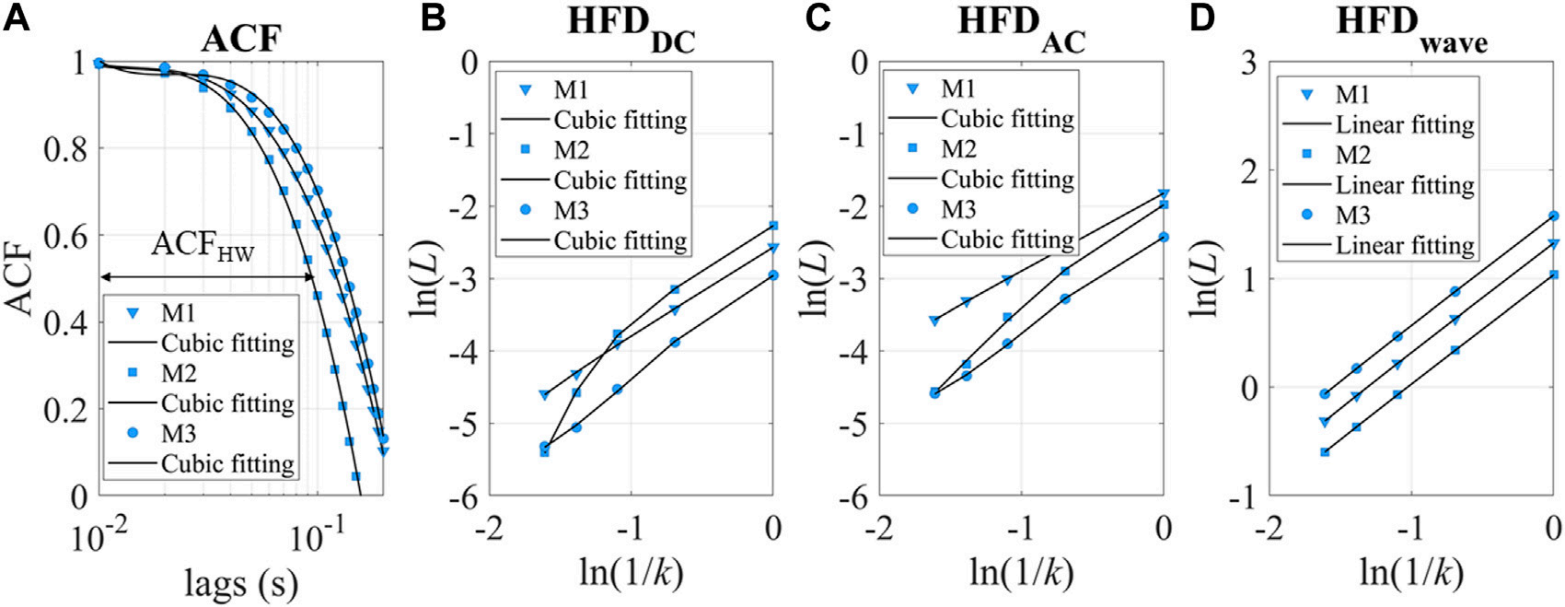
**(A)** ACF of three experimental measurements as examples. M1-3 refers to measurements 1–3. ACF_HW_ refers to the time lag taken to reach 0.5. **(B, C)** ln(*L*) *versus* ln(1/*k*). The intercept of the cubic fitting is used for HFD_DC_ and HFD_AC_ calculation. **(D)** ln(*L*) *versus* ln(1/*k*) for HFD_wave_ calculation.

We also introduced two main categories of parameters to describe the dynamic patterns: autocorrelation and fractal dimension. Autocorrelation Function (ACF) measures the correlation between data points in a time series and their preceding data points (Box et al., 2015; Tunnicliffe Wilson et al., 2015). It provides insights into the translation invariance of the signal across different delay times (τ). A commonly used measure derived from the ACF is known as ACF_HW_, representing the time when the ACF reaches 0.5, as depicted in Figure 5A.

Fractal dimension analysis is highly sensitive in uncovering hidden information within physiological time series (Higuchi, 1988; Kesić and Spasić, 2016a; Rubega et al., 2020). Most fractal measures require a long recording time of the signals (∼hours), which may not be suitable for BP estimation (Baumert et al., 2005). Since BP fluctuations occur on a shorter timescale (minutes), HFD proves to be a favorable choice for capturing the complexity and dynamic patterns in the data.

In this study, we used the HFD of PPG waveforms (HFD_wave_), baseline (HFD_DC_), and pulsatile amplitude (HFD_AC_) to measure complexity at different timescales. PPG signals could be simplified as time sequences *x*(1), *x*(2),…, *x*(N). *x* is sampled at 100 Hz for HFD_wave_, as a short-timescale fractal measure proposed by Cymberknop *et al.* (Cymberknop et al., 2011). HFD_DC_ and HFD_AC_ are calculated when *x* is sampled per cardiac cycle, representing longer timescale complexity (Colovini et al., 2019). From the starting time, a new self-similar time series is used to calculate curve length *L*_m_(*k*), as in Equation 7. *N* is the length of the original time series *x*, *m* is the initial time and *k* is the time interval. *int*[ $\left.\frac{N-m}{k}\right]$ is the integer part of the real number $\frac{N-m}{k}$ . In this study, *k*_max_ is set to 5.

$$L_{m}\left(k\right)=\frac{1}{k}[(\sum_{i=1}^{\mathrm{int}\left[\frac{N-m}{k}\right]}\left.\left|x\left(m+ik\right)-x\left(m+\left(i-1\right)k\right)\right|\right)\frac{N-1}{\mathrm{int}\left[\frac{N-m}{k}\right]k}\;;\;k=1,2,\ldots ,k_{\max}$$ (7)

*L*_m_(*k*) is averaged for all *m*. The mean value of the curve length *L*(*k*) is defined as

$$L\left(k\right)=\frac{1}{k}\sum_{m=1}^{k}L_{m}\left(k\right)$$ (8)

The ln(*L*(*k*)) and ln(1/*k*) relationship for DC and AC is sometimes nonlinear, as shown in Figures 5B,C. The fitting parameters contain rich information about hemodynamics. To describe the relationship between ln(*L*) and ln(1/*k*), a cubic fitting is employed, resulting in the following expression: ln(*L*) = a_0_+a_1_ln(1/*k*) +a_2_ln(1/*k*)^2^ + a_3_ln(1/*k*)^3^. Notably, the higher-order fitting coefficients (a_1-3_) demonstrated fractal property of the “active” stimuli at longer timescales, as demonstrated in Supplementary Figure S4. In this study, our focus is on examining the accumulation of “passive” responses, resulting in the usage of the intercept(a_0_) as HFD_AC_ or HFD_DC_. It is important to note that a_0_ is more closely related to stochastic signal fluctuation, while a_1_ represents the traditional definition of fractal dimensions. Conversely, we found that the linear slope of HFD_wave_ had enhanced robustness and correlated with the individual’s hemodynamic status. Therefore, the linear slope (a_1_) is used as the HFD_wave_ in our study.

### 2.6 Robustness of temporal patterns and their sensitivity to BP

Typically, fractal dimensions are calculated using longer signals (Baumert et al., 2005; Kesić and Spasić, 2016b). In our study, we chose 20 s to be the window size. It is necessary to evaluate the uncertainty of complexity measure calculation in this specific application. To address this, we undertook two approaches.

Firstly, we simulated a change in the coupling between the sensor and skin by multiplying the experimental PPG amplitudes by factors of 120% and 80%, respectively. We then recorded and analyzed the resulting variations in complexity measures. This evaluation allowed us to assess the sensitivity of the chosen measures to changes in the skin-sensor coupling coefficient.

Secondly, we estimated the error in slope(a_1_) and intercept(a_0_) estimation caused by cubic fitting of ln(*L*) *versus* ln(1/*k*) using experimental data. The errors were calculated by using variance-covariance matrix for the fitted coefficients (Seber, 1989). This analysis provided us with insights into the potential estimation errors caused by the fitting process.

As the goal of our study is to estimate the contribution of temporal patterns to blood pressure (BP) estimation, we evaluated their sensitivity to BP using the partial derivative ∂f/∂h . Here, *f* represents the chosen complexity measure, and *h* can be underlying hemodynamic parameters such as SV, *R* and *C*_1_. Measurements were divided into low and high CO variations according to their beat-to-beat CO fluctuations ( $\left|\partial CO/\partial t\right|)$ . The threshold is set to be the median of the CO variations.

### 2.7 Enhancing BP estimation performance with temporal features

To evaluate the impact of temporal complexities on BP, we constructed a straightforward Bayesian neural network(BNN) (Kesić and Spasić, 2016b). Morphological and temporal features, including HRV, were tested as standalone features and feature combinations. The resulting BNN performance may help understand the non-linear side of the relative contribution.

The BNN consisted of a single layer with 15 neurons. To address any imbalances in the input data and improve overall BP estimation performance, we utilized the EasyEnsemble technique (Liu, 2009). This technique effectively balances the data, leading to improved estimation accuracy. To ensure robust testing and training, we implemented a leave-one-subject-out procedure, allowing us to separate the training and testing data. In terms of the testing data, they were fitted and then calibrated using the first 10 data points. We assessed the MAP and PP under high and low CO fluctuation situations. Median absolute errors (MAE) and Pearson’s correlation coefficient (*r*) are used as indicators of accuracy and correlation. These evaluation measures provide valuable insights into the effectiveness of the BP estimation algorithm and its ability to accurately predict BP values.

Please be noted that this neural network structure or feature combination may not be the optimal for real-life deployment. The purpose is to showcase the added value of temporal features.

## 3 Results

This section presents the analysis of the *in silico* simulation results, which were further examined and verified using experimental data. The distributions of HFD and ACF are visualized, allowing for a thorough estimation of their respective contributions to BP. Additionally, comparisons with morphological features under both stable and unstable cardiac conditions were conducted to provide further insights.

### 3.1 Pulse simulation

**FIGURE 6:**
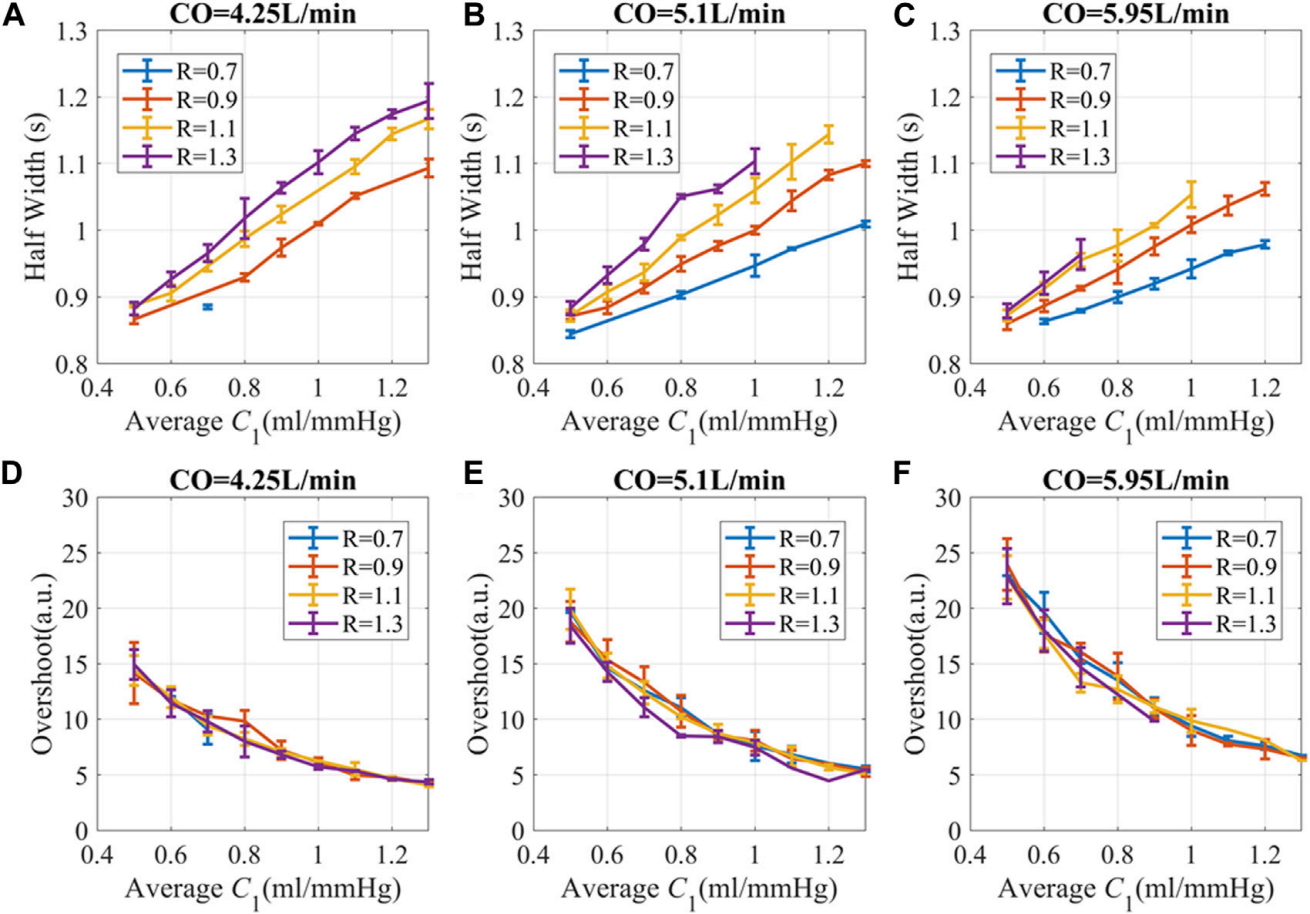
PPG recovery parameters *versus* hemodynamic status at different CO. **(A–C)** Half width (HW) of PPG response **(D–F)** Overshoot of PPG amplitude. The unit for *R* is “mmHg.s/ml”. The simulation data is presented as “mean ± SD” to accommodate the variations attributed to different *C*_2_ and *L*. Here, SD refers to the standard deviation of the data in each bin.

Since *C*_1_(t) changes rapidly during a cardiac cycle, to simplify the illustration, *C*_1_(t) is averaged and binned to suppress *C*_1_-profile-related fluctuations. To ensure a consistent comparison of amplitudes, a generalized transfer function was used to convert BP to PPG (Millasseau et al., 2000). We found that at a given peripheral resistance, PPG with higher *C*_1_ had a longer HW or slower recovery time after perturbation, as shown in Figures 6A–C. Lower peripheral resistance reduces the recovery time and narrows the HW differences for different *C*_1_. Smaller CO leads to longer recovery time and lower overshoot, which is probably due to lower BP and hysteresis. Generally speaking, subjects with stiffer blood vessels, lower peripheral resistance, and high CO had a more instantaneous response to stimuli. Subjects with very elastic blood vessels, high peripheral resistance, or low CO have prolonged responses, which may lead to complex overlap patterns.

The overshoot of PPG caused by the pulse stimulus is higher for subjects with lower *C*_1_ and high CO, as shown in Figures 6D–F. Since *C*_1_ becomes smaller with age (Brandfonbrener et al., 1955; Van Bortel and Spek, 1998), older subjects with hypertension are more likely to have high fluctuation of BP during the day, which is consistent with previous publications (Chobanian et al., 2003; Force et al., 2021; Guirguis-Blake et al., 2021).

Similar patterns exist for “mini” pulse simulations, except that PPG responses are much smaller. Pulse simulation with other hemodynamic conditions might be extrapolated from existing results. Another notable point is that all the possible hemodynamic combinations were used as long as the resulting BP is in the desired range. Experimental data showed overall higher *C*_1_ since the participants were young and healthy.

### 3.2 Continuous simulation

**FIGURE 7:**
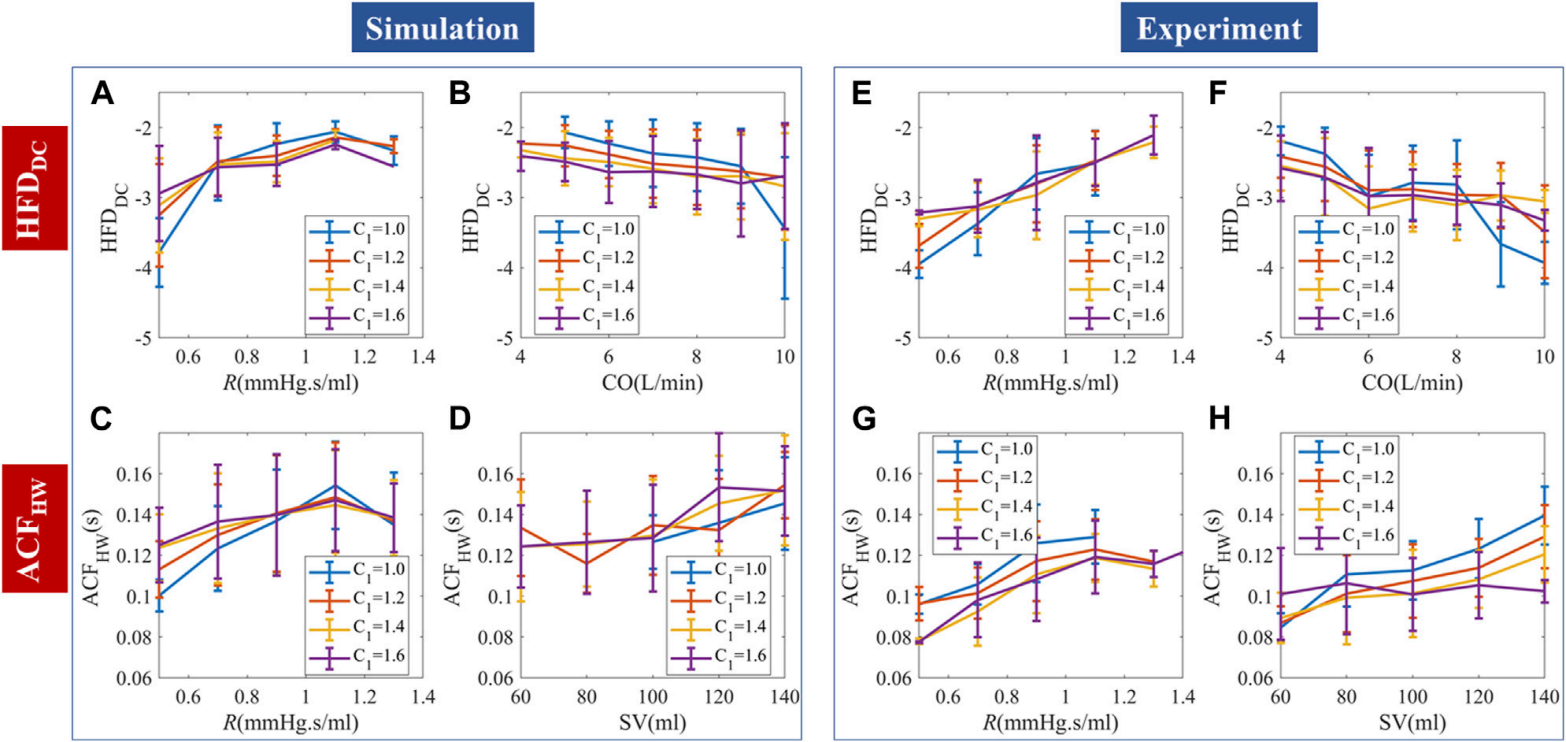
Comparison of continuous PPG simulation and experimental results. **(A–D)** Random stimuli caused complexities and their dependence on hemodynamic status. **(E–H)** Measured complexities and their dependence on hemodynamic status. The simulation and experimental data are binned and presented as “mean ± SD”.

Continuous simulation showed that temporal patterns could be generated by random stimuli, as in Figures 7A–D. Experimental results are similar but not the same as the simulation prediction, due to the autocorrelation patterns of stimuli, perturbation strength, and noise.

We used HFD_DC_ and ACF_HW_ to demonstrate the PPG temporal responses to continuous stimuli. HFD_DC_ showed a significantly positive correlation with *R*. Steiger’s z-test showed no statistical difference between the HFD_DC_-*R* slopes of the simulation and experiments. Experimental data had overall more negative $\partial {HFD}_{DC}/\partial CO$ slope compared to the simulation, but the slope is not smooth with locally positive gradients in the 6–8L/min subregion. The correlation between HFD_DC_ and *C*_1_ is non-significant in both simulation and experimental data.

For random stimuli, ACF_HW_ significantly and positively correlated with *R* and SV. Experimental data had significantly more positive $\partial {ACF}_{HW}/\partial R$ and $\partial {ACF}_{HW}/\partial SV$ slopes. We used SV instead of CO, because the correlation between ACF_HW_ and CO is positive but much weaker. Since the timescale of ACF_HW_ is small (∼0.1s), the inherent ACF dynamics likely correlate more with SV than CO. The correlation between ACF_HW_ and *C*_1_ changed with *R* in simulation, while experimental data showed a consistent negative correlation with *C*_1_. The difference is probably caused by *q*_in_-irregularity-induced phase lags. The minimum experimental ACF_HW_ (∼0.08s) is lower than the simulated ACF_HW_ (∼0.1s), probably due to the deviation from WK4 models caused by structural heterogeneity. The comparative analysis of the average slopes between temporal features and hemodynamics can be found in Supplementary Table S1. To enhance the quality of regression, a robust fitting approach was employed.

The relationship between HFD_AC_, HFD_wave_, and hemodynamics is shown in Supplementary Figure S5. For both simulation and experimental data, HFD_AC_ negatively correlated with *R* and positively correlated with CO. Experimental analysis showed similar trends with weaker *C*_1_ reliance. Experimental HFD_wave_ showed stratified but mixed correlations with *C*_1_. The correlation between HFD_wave_ and CO or *R* is also nonlinear, while simulations with random stimuli per heartbeat showed no corresponding trends. This result agreed with our hypothesis that HFD at a much shorter timescale (∼0.01s) may be caused by *q*_in_ irregularity and buildup of “mini” stimuli. Due to the high computational cost of adding random *q*_in_ irregularity and the difficulty of obtaining clinical *q*_in_ measurements, we think this explanation is plausible, but could not confirm this hypothesis at this stage.

### 3.3 Robustness of temporal patterns and their multi-dimensional mapping

To investigate the practical usage of temporal patterns in BP estimation, we tested their robustness to scaling factors. Three-dimensional distribution and sensitivity maps were generated, so that machine learning algorithms could use them as references.

#### 3.3.1 Sensitivity to PPG amplitude

**FIGURE 8:**
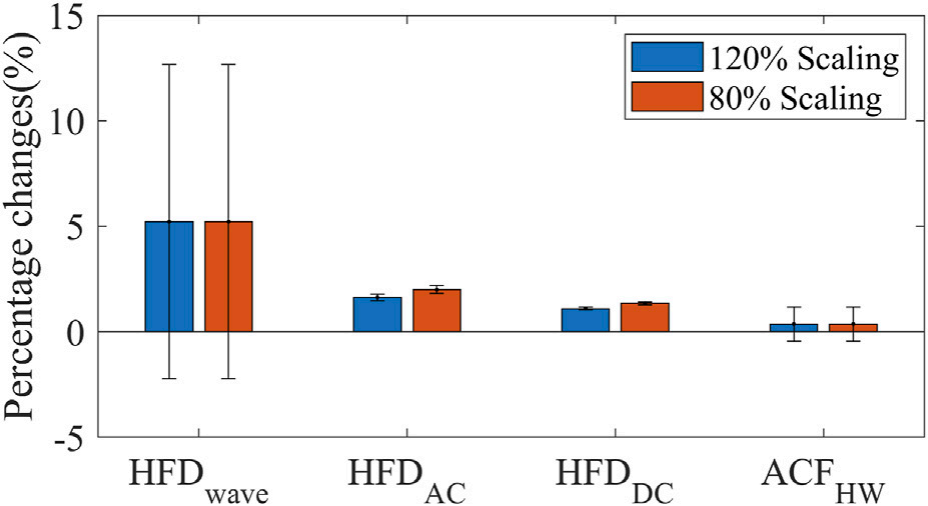
Scaling factor-induced HFD and ACF changes (mean ± SD).

The experimental PPG amplitudes were multiplied by 120% and 80% respectively to mimic the changed coupling between the sensor and skin. All the complexity measures are robust to PPG scaling factors, as shown in Figure 8. HFD_wave_ is the most influenced by the scaling factors. But the magnitude (5%) is still much smaller than the disturbances (±20%).

#### 3.3.2 Influence of data segment length

The intercept(a_0_) of HFD calculation reveals a small relative uncertainty even with a signal length of 20 s. However, the uncertainty of the slope(a_1_) is more influenced by the signal length, showing stabilization around 100 s. It is worth noting that the intercept and slope of the ln(*L*) *versus* ln(1/*k*) values can fluctuate by 50% and 200% respectively, during a 5-min measurement. Thus, the uncertainty of the HFD calculation remains relatively small.

In this study, our primary focus is on the accumulation of delayed cardiovascular response to stimuli, which is more related to the intercept of the HFD calculation. Supplementary Figure S4 demonstrates that the higher-order fitting coefficients of ln(*L*) *versus* ln(1/*k*) exhibit a strong correlation with the corresponding fractal dimensions of stroke volume (SV) only when signal lengths reach 100 s or longer. Hence, it is crucial that future studies, which incorporate a closed-loop model and consider CO or SV complexities, utilize longer signal lengths to ensure accurate and reliable calculations. This would enable a more comprehensive understanding of the relationship between the higher-order coefficients, fractal dimensions, and physiological parameters.

#### 3.3.3 Distribution of complexity measures and their sensitivity to hemodynamics

**FIGURE 9:**
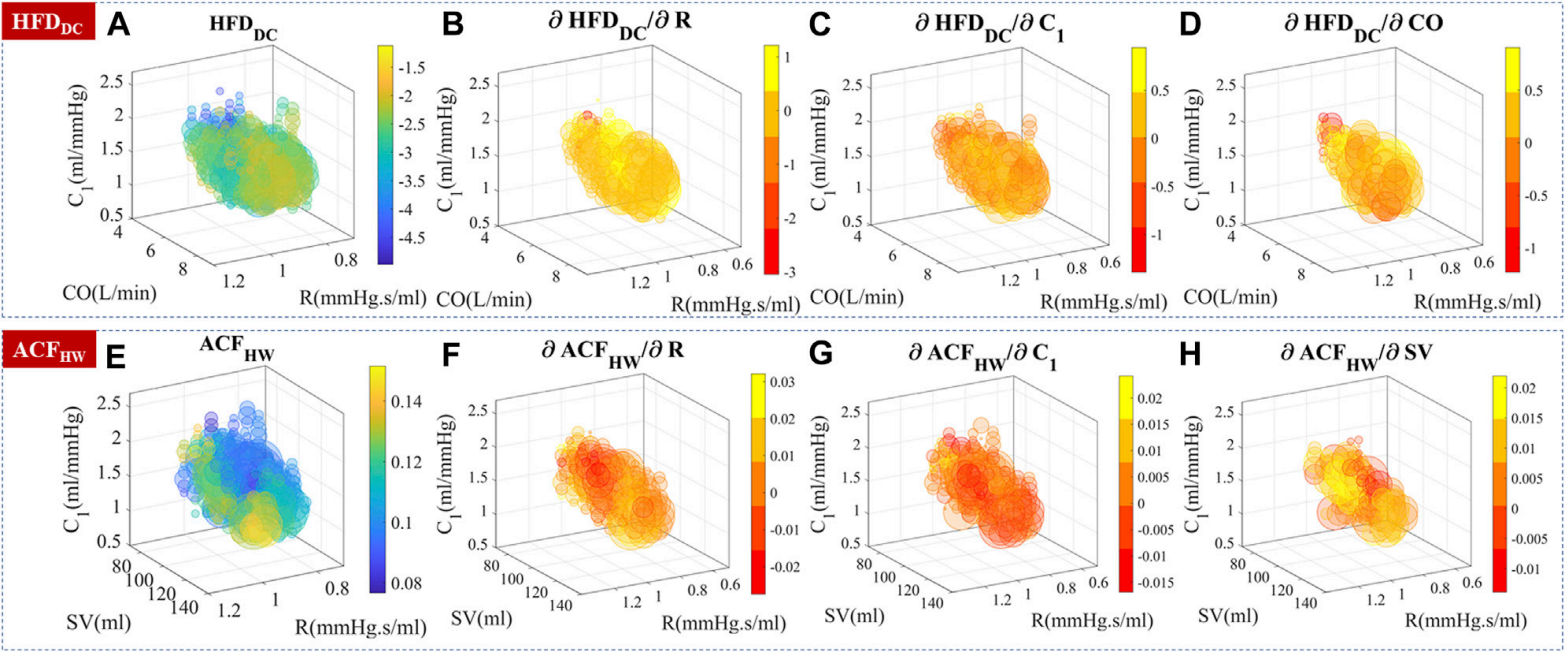
Experimental temporal complexity measure distributions and their sensitivity to CO, *R,* and *C*_1_. The size of the bubble represented the relative number of measurements. The color represented the complexity measure or gradient value. **(A–D)** HFD_DC_ distribution and its sensitivity to hemodynamic parameters. $\partial {HFD}_{DC}/\partial CO$ is locally positive in the 6–8 L/min subregion. **(E–H)** ACF_HW_ distribution and its sensitivity to hemodynamic parameters.

Experimental data were used to generate a three-dimensional map of complexity measures based on CO, *R,* and *C*_1_, as shown in Figure 9. To ascertain the sensitivity of complexity measures to hemodynamic changes, the partial differentiation technique was employed while controlling the effects of the remaining variables. Although the presence of autocorrelation in stimuli, perturbation strength, and noise may cause non-smooth sensitivities, their distribution and differentiability still provide valuable information for analysis.

Knowing the underlying hemodynamic properties of the subject would help build more robust and accurate BP estimation algorithms. Self-similarity and stochastic patterns could encode the hemodynamics-related information in PPG temporal series, which may help stabilize BP estimation and improve the overall performance.

### 3.4 Contribution to BP: Linear correlation

**FIGURE 10:**
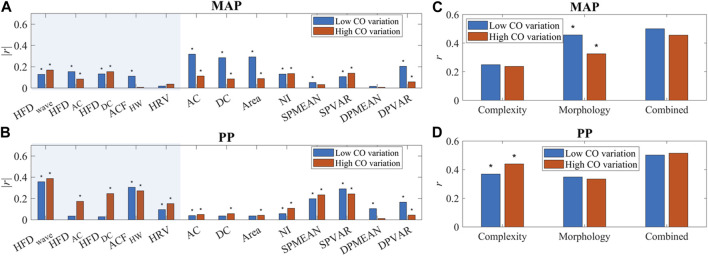
**(A, B)** The correlation of PPG features and BP (MAP and PP). “*” indicated a significant difference between groups. Temporal features are in the shaded area. **(C, D)** Correlation of BP (MAP and PP) estimation and reference with complexity features only, morphology features only, and combined features. “*” indicated a significant difference between low and high CO variation.

As shown in Figure 10, temporal complexity features are less influenced by CO fluctuation, and some even had increased correlation at high CO variation. Most morphological features had a significantly decreased or small correlation with MAP at high CO variation. A simple multiple linear regression algorithm was built with these features. The PCC of estimated MAP and PP with reference is shown in Figures 10C,D. BP estimation performance is not affected if morphological features and temporal features are combined, while the morphology-only algorithm has a significantly worse MAP estimation performance at higher CO fluctuations.

### 3.5 Evaluation of nonlinear correlations with BP

Nonlinearity exists in the PPG-BP relationship. To further investigate the impact of different features on BP estimation performance, a BNN algorithm was employed, as described in Section 2.6. Notably, the combination of morphological features, HRV, and complexity measures such as HFD and ACF exhibited the highest correlation with the reference for BP prediction, although it did not yield the lowest MAE. Upon closer examination, it was found that the inclusion of complexity measures introduced more outliers compared to the morphology only method, as shown in Supplementary Figure S6, S7. This finding can be attributed to the inherent uncertainties associated with HFD calculations, which should be carefully considered. To address this issue, implementing a thorough outlier removal or quality check procedure would prove beneficial.

The addition of HRV played a significant role in scenarios with high CO fluctuations. However, it was observed that the incorporation of HRV negatively impacts BP estimation performance when CO fluctuations are low. These findings underscore the importance of considering the specific context and characteristics of the dataset when selecting and combining features for BP estimation. Table 3 Table 4.

**TABLE 3:** Uncertainty of HFD calculation caused by length selection.

	HFD of DC		HFD of AC	
	Intercept(a_0_)	Slope (a_1_)	Intercept(a_0_)	Slope (a_1_)
20	0.60%	20.0%	0.42%	27.0%
60	0.31%	9.6%	0.23%	13.5%
100	0.27%	8.0%	0.19%	10.8%

**TABLE 4:** Calibrated BP estimation by BNN.

		High CO fluctuation		Low CO fluctuation	
		MAP	PP	MAP	PP
Correlation coefficient (*r*)	M	0.50	0.61	0.58	0.66
	M + HRV	0.62	0.66	0.42	0.57
	M + HRV + complexity	0.65	0.71	0.61	0.74
MAE (mmHg)	M	4.47	6.78	4.23	6.82
	M + HRV	5.12	8.08	5.18	8.38
	M + HRV + complexity	4.97	7.45	4.61	7.51

## 4 Discussion

### 4.1 Novelty of the study

Single-site PPG-based BP estimation has raised a lot of interest in both academia and the industrial world. Previous studies showed that PPG morphological information may not be enough to estimate BP, but the dynamic process is too complex to build a precise model. Machine learning algorithms such as LSTM produced better results. But it is difficult to know the exact mechanism.

In this study, we designed a novel *in silico* simulation to provide insight into the hemodynamic process. Pulse simulations showed that CO, *R*, and *C*_1_ are the main determinants of prolonged PPG fluctuations. Continuous simulation with random stimuli confirmed that the buildup of prolonged vascular responses could generate certain stochastic patterns, which had a strong dependence on hemodynamics. Experimental data agreed well with the prediction. Real-life stimuli could have different levels of autocorrelation and perturbation strengths. The multi-scales and nonlinearity of complexity should be utilized to capture the hemodynamics-related information.

In addition to providing more information about hemodynamics, HFD, and ACF features are more robust. For repetitive measurement, the coupling coefficient of skin and sensor, and the sensor location difference may cause large errors in PPG amplitude measurement. Complexity patterns are less sensitive to PPG amplitude, which may help stabilize BP estimation. At unstable conditions when CO variation is higher, more and more information go into temporal complexity features. The contribution of morphological features to BP significantly decreased. BP estimation algorithms could only have the same performance when temporal features were added, showing the inherent information flow when cardiac stability changes.

### 4.2 Comparison with previous studies

Very few studies have used explicit stochastic temporal features to estimate BP (Hosanee et al., 2020). Colovini, T. *et al.* reported that HFD of the continuous DBP recordings had a significantly positive correlation with average DBP in hypertensive patients (Colovini et al., 2019). Cymberknop *et al.* found that HFD of arterial pressure morphology decreased with increasing blood flow, which correlated with arterial stiffness (Cymberknop et al., 2011). Gomes, R. *et al.* reported acutely decreased HFD of heart rate after exercise (Gomes et al., 2017). The fractal dimension of signals as short as 50 heartbeats is useful (Peña et al., 2009). To our knowledge, ACF has not been studied as a contributing factor to BP estimation. Although HRV is a well-studied dynamic feature, its impact on BP improvement primarily relates to the assessment of neurological activity (Mejía-Mejía et al., 2020a; Mejía-Mejía et al., 2020b; Mejía-Mejía et al., 2022), representing the "active” stimuli and not encompassing all aspects of the dynamic process.

In our study, we gave explicit disclosure about the temporal PPG patterns and their potential physiological meanings. Their relative contribution to BP depended on hemodynamic properties. By mapping the distribution of temporal complexity features, the application scope could be defined.

### 4.3 Limitations of the study

The database we used contained 40 healthy subjects. Most of them were young and healthy. The same procedure should be tested in datasets with wider coverage of subjects. Human beings are not perfect stochastic systems, and the approximation of HFD and ACF calculations may not be accurate enough. Although sex is unlikely to influence PPG-BP correlation, its role in cardiovascular health deserves to be explored further with PPG technology. Finally, we used estimated blood vessel compliance and peripheral resistance to generate the complexity feature distribution map. Validation from medical ultrasound and total peripheral resistance (TPR) measurements should be necessary.

We proposed a plausible explanation of the stochastic patterns in the PPG signal. The simulation results agreed with experimental observations and previous publications. However, the exact origin should be explored further, and the cardiovascular modeling should be more detailed to account for the closed-loop interaction, vascular tree structure, flow distribution, cardiac activity variation, *etc.*

### 4.4 Suggestions for future work

Temporal complexities contain rich information about hemodynamics. Our study only showed a fraction of its potential due to limited space. Previous studies mostly used HFD of BP instead of PPG signals. The impact of *P-V* translation and measurement location on the HFD of PPG should be fully investigated. For example, simulating PPG signals in peripheral areas *versus* within the arterial network may yield distinct results due to variations in the volume of arterial blood within the tissues. It would be intriguing to extend the application of the time-dependent model to incorporate multi-site PPG measurements, allowing for a more comprehensive investigation. We have shown that the nonlinearity of ln(*L*) and ln(1/*k*) relationship contained stimuli information. The role of other fitting coefficients should be thoroughly investigated. The incorporation of these coefficients will further improve the BP estimation accuracy. We found a significant contribution of HFD_wave_ to BP. However, its correlation with hemodynamic parameters is nonlinear. Collection of clinical *q*_in_ and corresponding simulations should be done to elucidate its influence on HFD_wave_.

It is possible that we only found one source of temporal PPG complexity. A more detailed simulation including cardiovascular tree structure should be carried out to confirm the origin of the fractal pattern. In addition to SV or CO variations, the influence of breathing should be taken into account, considering its impact on the parasympathetic and sympathetic nervous systems. Furthermore, conducting simulations with different patterns of HRV may provide valuable insights. By using a larger dataset and employing finer grids, more accurate and comprehensive heatmaps of temporal features in relation to BP can be generated. Incorporating these adjustments and considerations will contribute to a more holistic understanding of the intricate relationship between PPG and BP.

We employed a simple BNN to estimate BP, which, although explicit, may not yield optimal performance. Moving forward, it is crucial for future studies to consider the temporal properties of the algorithm and fine-tune parameters in alignment with the specific context, such as stimuli strength, hemodynamic status, and physiological constrains related to temporal interactions. These refinements will contribute to enhancing the performance and accuracy of BP estimation algorithms.

Although we obtained decent BP estimation performance from single-site PPG alone, it is important to note that relying solely on single-site PPG or a simple combination of PPG and ECG may not provide sufficient information for reliable BP estimations (Mieloszyk et al., 2022; Mukkamala et al., 2023). Therefore, adopting a multi-modal approach that integrates multiple sensing modalities is crucial to enhance the reliability of the algorithm. This entails extracting valuable information from each modality and evaluating their respective contributions. By improving data integration and analysis across various physiological measurements, we can achieve more accurate and robust BP estimation outcomes.

## 5 Conclusion

Signal fluctuation is not merely a nuisance but also valuable information. In this study, we have demonstrated that the temporal complexity patterns of PPG are correlated with hemodynamic status and make a substantial contribution to BP estimation, particularly in the presence of high CO variations. The integration of these temporal complexity features has the potential to enhance the accuracy and interpretability of single-site PPG-based BP estimation methods, thereby facilitating the development of more advanced algorithms in the future.

## Data Availability

The original contributions presented in the study are included in the article/Supplementary Material, further inquiries can be directed to the corresponding authors.
