# Supplementary material for: Temporal complexity in photoplethysmography and its influence on blood pressure
Source: Front Physiol. 2023 Aug 31;14:1187561. doi: 10.3389/fphys.2023.1187561 (PMC10513039; doi:10.3389/fphys.2023.1187561)
Supplement: Supplementary file 1 [file DataSheet1.pdf]

Supplementary Material

Temporal Complexity in Photoplethysmography and Its Influence on Blood Pressure

1 Supplementary Figures

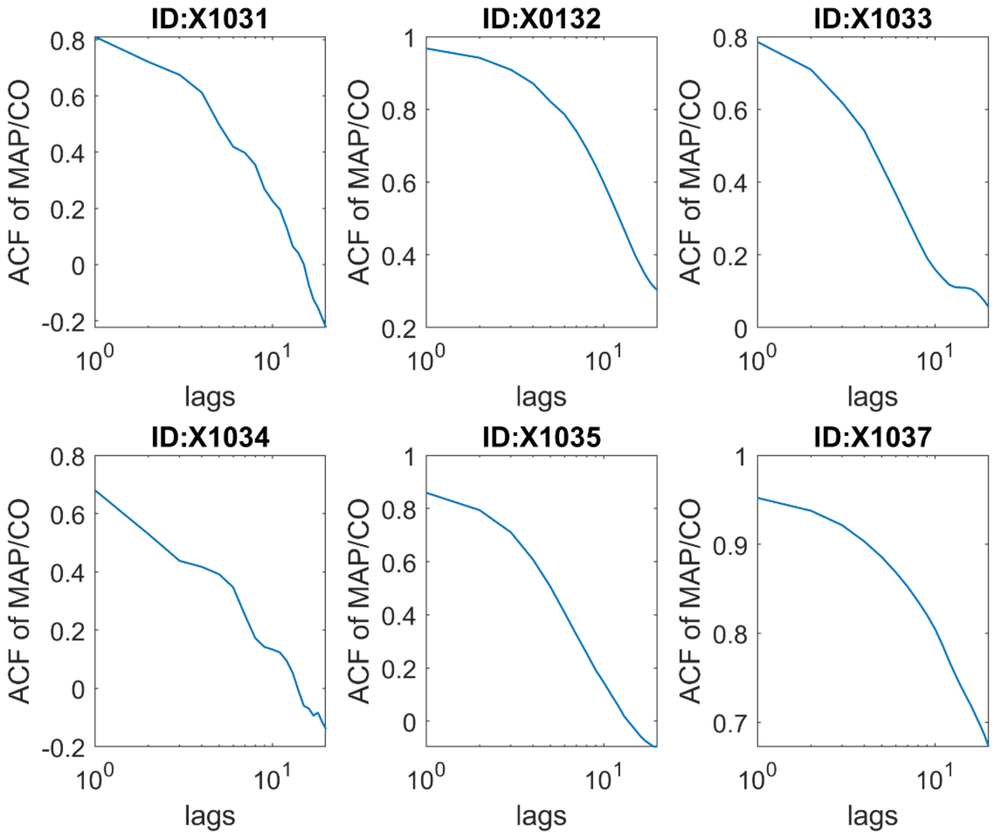

Supplementary Figure 1. Examples of MAP/CO autocorrelation from experimental measurements. MAP/CO was calculated per cardiac cycle and linearly correlated with *R*. MAP is the mean arterial pressure, and CO is stroke volume multiplied by heart rate.

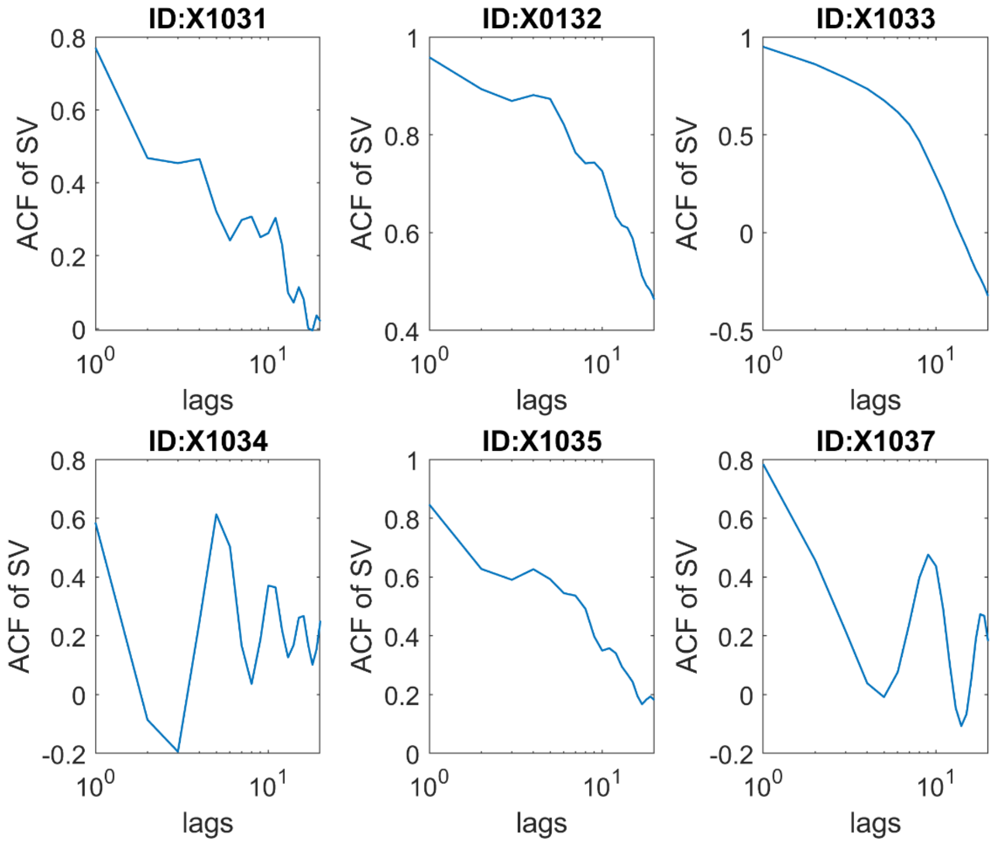

Supplementary Figure 2. Examples of stroke volume (SV) autocorrelation from experimental measurements. SV was calculated per cardiac cycle.

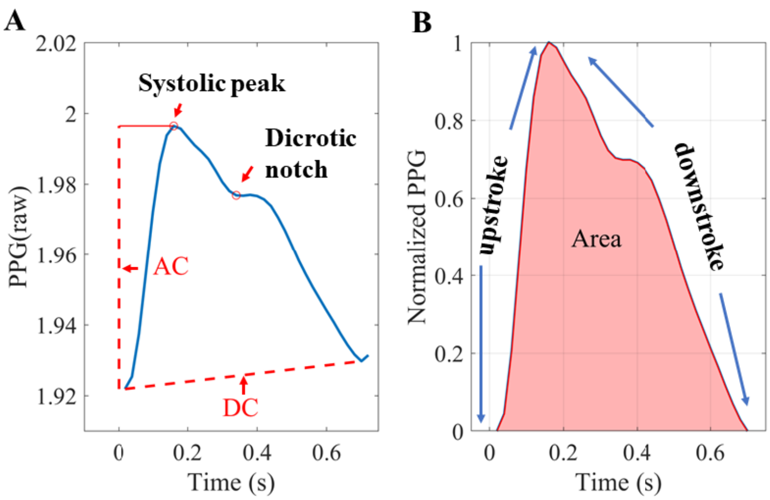

Supplementary Figure 3. Illustration of PPG waveform and used morphological features (A) Definition of AC, DC, systolic peak, and dicrotic notch; (B) Normalized PPG waveform and its upstroke and downstroke slopes. "Area" was defined as the area under the curve.

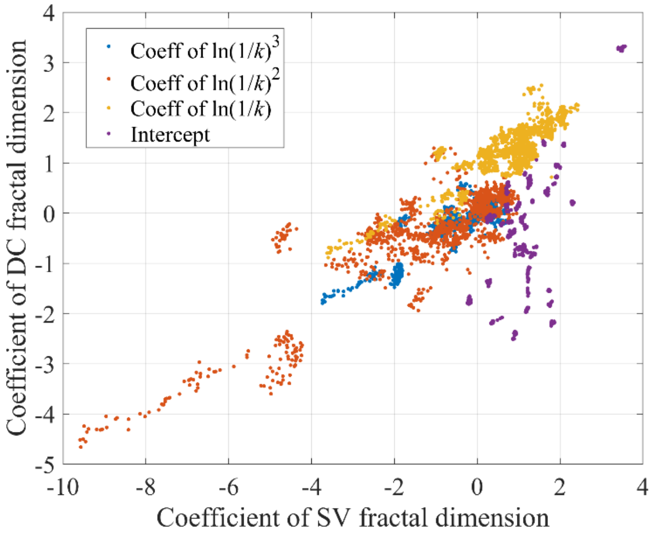

Supplementary Figure 4. Illustration of long-term fractal dimension components (N~100 cardiac cycle). Each window moved 10s relative to the preceding window. A total of 1334 data points were generated. The higher-order fitting coefficients of ln(L) versus ln($1/k$) were highly correlated (r~0.85). The correlation disappeared if a shorter window was used (N~20 cardiac cycles). The intercept of a

20-cardiac cycle window was used to investigate the "passive" and faster cardiovascular response with random SV and *R* stimuli.

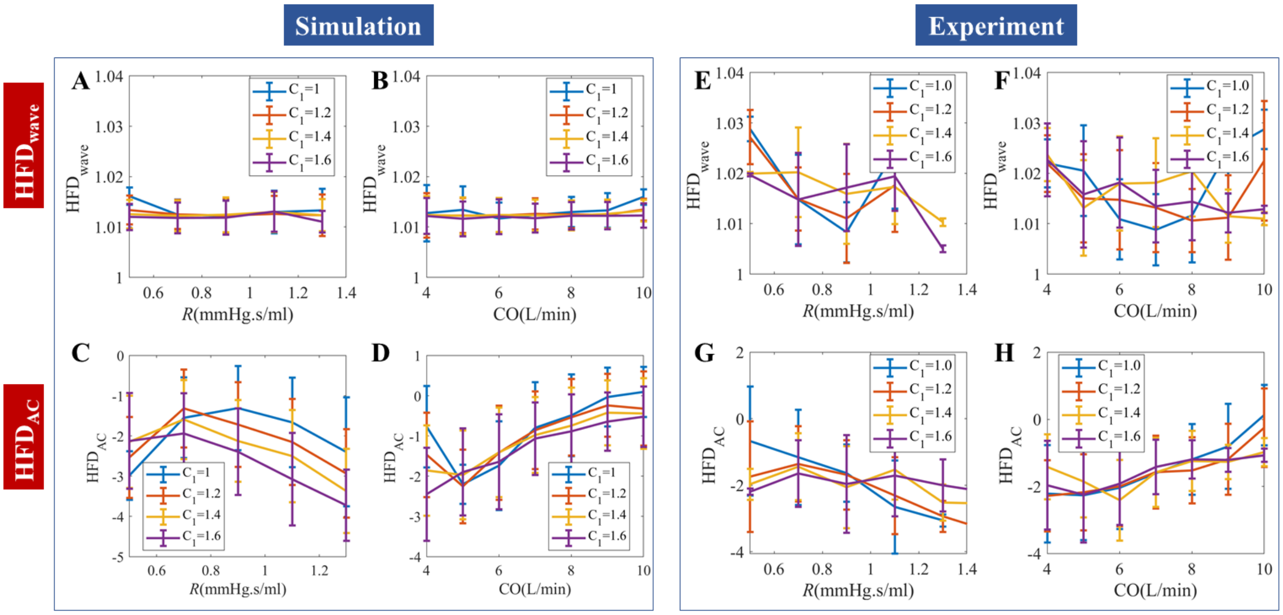

Supplementary Figure 5. Comparison of continuous PPG simulation and experimental results. (A-D) Random CO and *R* stimuli caused complexities (HFD_{wave} and HFD_{AC}) and their dependence on hemodynamic status. Since HFD_{wave} was calculated at a much shorter timescale(~0.01s), the SV and *R* stimuli didn't produce any meaningful HFD_{wave} patterns. (E-H) Measured complexities and their dependence on hemodynamic status.

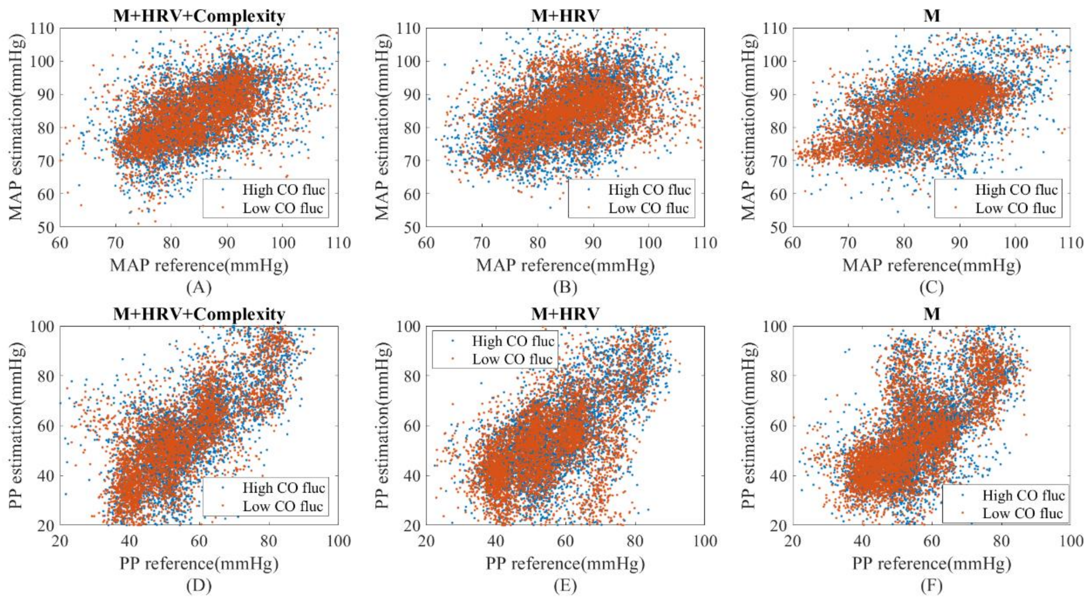

Supplementary Figure 6. (A-C), MAP estimation depending on the feature combinations. (D-F), PP estimation depending on the feature combinations. "High/low CO fluc" refers to data segments with ΔCO higher or lower than the median of ΔCO across all data segments. ΔCO refers to the absolute value of CO variation between adjacent data segments.

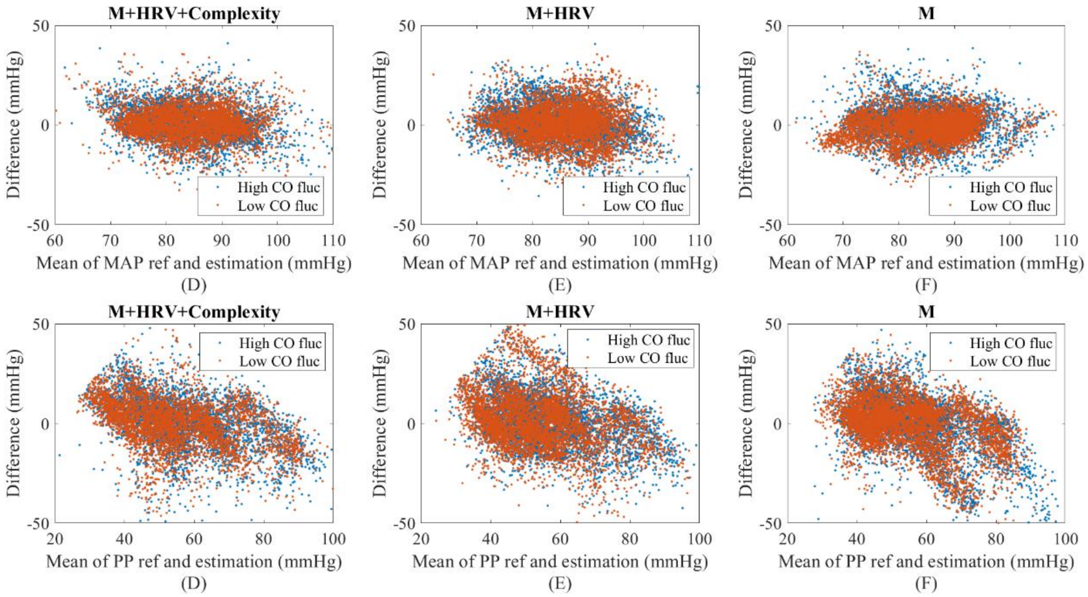

Supplementary Figure 7. (A-C), The bland-Altman plot of MAP estimation depending on the feature combinations. (D-F), The bland-Altman plot of PP estimation depending on the feature combinations. "ref" means reference value.

2 Supplementary Tables

Supplementary Table 1. Comparison of major complexity measures and hemodynamic parameters

	Simulation				Experiment			
	R		CO		*R*		CO	
	Offset	Slope (ml/mmHg/s)	Offset	Slope (min/L)	Offset	Slope (ml/mmHg/s)	Offset	Slope (min/L)
**HFD_{DC}**	-3.78	1.62	-1.58	-0.14	-4.45	1.84	-2.04	-0.13
**HFD_{AC}**	-0.88	-1.12	-3.54	0.23	-0.54	-1.37	-3.17	0.22
**HFD_{wave}**	1.01	-0.0015	1.01	0.00	1.02	-0.01	1.02	-0.00
	R		SV		*R*		SV	
	Offset	Slope (ml/mmHg/s)	Offset	Slope (/100ml)	Offset	Slope (ml/mmHg/s)	Offset	Slope (/100ml)
**ACF_{HW}**	0.12	0.02	0.11	0.03	0.09	0.07	0.08	0.05
